# Disparities of Food Availability and Affordability within Convenience Stores in Bexar County, Texas

**DOI:** 10.1155/2013/782756

**Published:** 2013-07-01

**Authors:** Matthew Lee Smith, T. S. Sunil, Camerino I. Salazar, Sadaf Rafique, Marcia G. Ory

**Affiliations:** ^1^Department of Health Promotion and Behavior, College of Public Health, The University of Georgia, 330 River Road, 315 Ramsey Center, Athens, GA 30602, USA; ^2^Department of Health Promotion and Community Health Sciences, School of Rural Public Health, Texas A&M Health Science Center, TAMU 1266, College Station, TX 77843, USA; ^3^Department of Sociology, University of Texas at San Antonio, One UTSA Circle, San Antonio, TX 78249, USA; ^4^Department of Quality and Outcomes, University Health System-Ambulatory Services, 701 S. Zarzamora, MS 41-5, San Antonio, TX 78207, USA

## Abstract

The American Diabetes Association (ADA) recommends healthful food choices; however, some geographic areas are limited in the types of foods they offer. Little is known about the role of convenience stores as viable channels to provide healthier foods in our “grab and go” society. The purposes of this study were to (1) identify foods offered within convenience stores located in two Bexar County, Texas, ZIP Codes and (2) compare the availability and cost of ADA-recommended foods including beverages, produce, grains, and oils/fats. Data were analyzed from 28 convenience store audits performed in two sociodemographically diverse ZIP Codes in Bexar County, Texas. Chi-squared tests were used to compare food availability, and *t*-tests were used to compare food cost in convenience stores between ZIP Codes. A significantly larger proportion of convenience stores in more affluent areas offered bananas (*χ* ^2^ = 4.17, *P* = 0.003), whole grain bread (*χ* ^2^ = 8.33, *P* = 0.004), and baked potato chips (*χ* ^2^ = 13.68, *P* < 0.001). On average, the price of diet cola (*t* = −2.12, *P* = 0.044) and certain produce items (e.g., bananas, oranges, tomatoes, broccoli, and cucumber) was significantly higher within convenience stores in more affluent areas. Convenience stores can play an important role to positively shape a community's food environment by stocking healthier foods at affordable prices.

## 1. Introduction

Well-known risk factors for developing chronic disease include being overweight or obese, being physically inactive, and having poor nutritional habits [1, 2]. Medical costs associated with being overweight (i.e., body mass index [BMI] of 25 to 29.9 kg/m^2^) and obese (i.e., BMI greater than 30 kg/m^2^) account for almost 10% of the total U.S. healthcare expenditures (estimated at $147 billion), of which approximately half are paid by Medicaid and Medicare [3–5]. Relative to their non-Hispanic white counterparts, Hispanic individuals are disproportionately burdened by chronic health conditions related to obesity including heart disease, stroke, hypertension, and diabetes [6–9]. Obesity- and disease-related disparities among Hispanic individuals have been related to lower socioeconomic status, less desirable lifestyle behaviors, less frequent healthcare utilization, and environmental factors impacting access to healthful resources and services [10].

While socioenvironmental factors are generally recognized as contributors to health status [11], studies about the food culture of certain communities have become the focus of research studies to identify areas considered to be “food deserts” [12]. Although food deserts traditionally indicate areas where healthy foods are not available to local residents [12], few studies specifically examine the availability and price of recommended healthy foods based on specific types of food outlets. Even fewer studies compare community-based food culture by the residential affluence, ethnic composition, or risk related to specific disease conditions.

A variety of initiatives have assessed food culture by conducting food outlet audits to identify the availability, affordability, and quality of common food stuffs [13–16]. These audits have been primarily conducted in grocery stores or supermarkets in an attempt to identify food culture as it pertains to health indicators in areas of varying affluence [17]. Food access remains an issue within less affluent and rural communities where food sources are scarcer. A study by Morland and colleagues [18] examined the role of food environment in recommended dietary intake among residents in 208 U.S. census tracts and found that the fruit and vegetable consumption among residents increased with the number of food outlets. These researchers also found that census tracts with large non-minority populations were five times more likely to have a supermarket compared to census tracts with a large minority population. Additional studies have similarly identified that economically disadvantaged communities have less access to supermarkets that offer affordable healthy food choices [13, 19–24], which leads these communities to become dependent on other food outlet types.

While studies frequently report the inventory of grocery stores, few specifically focus on the role of convenience stores in the food environment. It should be of no surprise that grocery stores stock a large variety of healthy and unhealthy foods because of their size and need to meet the purchasing demands of the population. However, in rural and economically underserved communities, grocery store locations are fewer and more dispersed, which makes the reliance on convenience stores for obtaining food greater in these areas. The presence of convenience stores is often more pronounced in economically underserved communities [23, 25], which emphasizes the importance that they provide affordable foods suitable to sustain the dietary needs of the surrounding community [26–28]. While convenience stores offer an alternative to grocery stores, the products may be priced higher and of lesser quality [20]. Therefore, access to healthy and affordable food is likely related to the type of food outlets in a particular community, not solely the number of outlets. With the reliance on convenience stores to provide basic nutritional needs, the expectation to carry healthy foods may be less. Although, for residents in areas with limited food outlets, convenience stores may provide the only opportunity to obtain healthy foods; and, when unavailable, the risk for obesity and related conditions may escalate.

As defined by the American Diabetes Association (ADA), healthy diets should consist of low-fat, high fiber foods including fruits, vegetables, and whole grains [29]. Additionally, the ADA recommends limiting the consumption of dairy fats, sweetened beverages, and fried foods.

Utilizing food recommendations outlined by the ADA, this study aims to (1) identify ADA-recommended foods including beverages, produce, grains, and oils/fats offered within convenience stores located in two Bexar County, Texas ZIP Codes and (2) compare the availability and cost of these ADA-recommended foods. Recognizing the vast socioeconomic and ethnic composition differences by area in Bexar County, Texas, two ZIP Codes were purposively selected for comparison: one more affluent ZIP Code with more non-Hispanic white residents and less diabetes prevalence, one less affluent ZIP Code with primarily Hispanic residents and more diabetes prevalence. 

## 2. Materials and Methods

### 2.1. Audit Procedures

During the spring of 2010, sociology students from the University of Texas at San Antonio (UTSA) enrolled in a health disparities course, and undergraduate nursing students from the University of Texas Health Science Center at San Antonio (UTHSCSA) enrolled in a community health course participated in data acquisition regarding healthy food choices in targeted areas of the city. Course descriptions included a critical analysis of historical, political, economic, social, cultural, and environmental conditions that have produced health disparities for racial and ethnic minorities. Therefore, an innovative teaching initiative targeting community health improvement was employed with these students to provide them with a more robust understanding of the environmental determinants of health disparities. To complement course content with practical experience, students engaged in field audits to gather and assess the nutritional food environment based in two distinct geographic areas (ZIP Codes) of the city. These field audits afforded the students with an opportunity to develop methodological skills in community-based research.

Working collaboratively in this educational initiative, institutional partners included UTSA, a four-year public university; UTHSCSA, a four-year medical school involving more than 100 affiliated hospitals, clinics, and health care facilities in South Texas; and the Texas Diabetes Institute, a member of University Health System, a clinical training partner, and a major urban safety net hospital system located in Bexar County, Texas.

### 2.2. Site Selection

For comparison purposes, site selection for nutritional food environment assessment was based on clinic visit data provided by the Texas Diabetes Institute that identified the highest and lowest areas of the city with adults clinically diagnosed with diabetes. This resulted in the selection of ZIP Codes 78207 (ZIP A; *N* = 55, 514 residents) and 78240 (ZIP B; *N* = 51, 111 residents). These ZIP Codes also reflected differing median household incomes and percentages of ethnic minorities.

Convenience store selection for both study ZIP Codes was obtained from the 2010 San Antonio area phone directory and from a business listing provided by the San Antonio Area Chamber of Commerce based on the U.S. Securities and Exchange Commission Standard Industrial Classification (SIC). A convenience store was defined as a small, higher-margin store offering a limited selection of staple groceries, nonfoods, and other convenience food items (e.g., ready-to-eat foods). The store may or may have not sold gasoline [30, 31]. A total of 37 retail convenience stores (SIC 5412) were identified in the two ZIP Codes of interest. Seven convenience stores were omitted from analyses because they were no longer in operation at the time of the study. Two convenience stores refused to participate in the study. Therefore, of the 37 convenience stores originally identified, 28 were included in in the analytic sample for this study.

### 2.3. Instrument and Measures

Data were collected by students who were trained to use the nutrition environment measures assessment, a standardized tool used to identify neighborhood needs regarding access to healthy foods [32]. Students visited convenience stores in both ZIP Codes to assess the availability (i.e., presence) and cost (i.e., price) of eight food groups: nonfat/low-fat milk, fruits, vegetables, low-fat meat, frozen foods, low-sodium foods, 100% whole wheat bread, and low-sugar cereals. Items in the instrument were standardized by brand, type, and size. 

 The previously validated observational tool is comprised of 10 measures that assess availability of food choice options (i.e., healthy versus unhealthy) as well as the cost (i.e., price) and quality of food products such as milk, produce (fresh fruits and vegetables), ground beef, hot dogs, frozen dinners, baked goods, beverages (soda/juice), whole grain bread, baked chips, and cereal [32]. Developed as part of the nutrition environment measures survey (NEMS), the NEMS-S was used to measure food availability in a variety of store types. The instrument's reliability was previously tested in 85 stores located in Atlanta, Georgia. Both interrater reliability and test-retest reliability were high for all food items examined [32]. Additional details about auditor training and orientation as well as a copy of the convenience store survey can be found on the NEMS website [32].

As previously mentioned, the food-related variables examined in this study were based on the ADA recommendations. Foods were grouped into the following categories: beverages, fruits and vegetables, grains, and liquid oils. Healthy and unhealthy food options for each category were included, with the exception of fruits and vegetables. Availability of food items was measured as whether or not the convenience store had the item in stock (i.e., available for purchase). Affordability of food items was measured as the selling price of the item (i.e., measured in U.S. dollars [USD]).

### 2.4. Data Analysis

All statistical analyses for this descriptive study were performed using SPSS (version 18). Frequencies were generated for all variables of interest. Study variables were then compared by the convenience stores' ZIP Code (i.e., 78207, 78240) using Pearson's chi-square tests for categorical variables (food availability) and independent sample *t*-tests for continuous variables (food affordability).

## 3. Results

Table 1 provides details about the social and health characteristics of the population located in ZIP Codes relative to Bexar County. A larger proportion of individuals residing in ZIP Code 78207 (ZIP A) were Hispanic (90.9%) compared to 47.7% of residents in ZIP Code 78240 (ZIP B) (i.e., compared with 54.4% of Bexar County residents and 32.0% of Texas residents). A larger proportion of individuals residing in ZIP A had less than a high school education (47.3%) compared to 7.6% of residents in ZIP B (i.e., compared with 27.1% of Bexar County residents and 20.0% of Texas residents). On average, residents in ZIP A had lower median household incomes ($20,120 USD) compared to residents of ZIP B ($39,630 USD) (i.e., compared with Bexar County [$38,330 USD] and Texas [$39,930 USD]). No substantial differences existed between study ZIP Codes by age-, diabetes-, or heart disease-related deaths. However, when comparing the presence of food outlets by ZIP Code, ZIP A had a larger number of convenience and grocery stores (23 and 14, resp.) compared to ZIP B (14 and 4, resp.).

A total of 28 convenience stores were inventoried for food availability and price within two ZIP Codes, 78207 and 78240.  Table 2 describes the availability of beverages, fruits and vegetables, grains, and liquid oils (chips) at convenience stores within study ZIP Codes. Nineteen convenience stores were assessed in ZIP Code 78207 (67.9%), and nine convenience stores were assessed in ZIP Code 78240 (32.1%).

In terms of healthier beverage availability, 100% of convenience stores sold diet cola (compared to 100% that sold regular cola), 10.7% sold lower fat milk (compared to 59.1% that sold 2% milk), and 71.4% sold fruit juice with no sugar added (compared to 39.3% that sold juice drink with sugar added). In terms of produce, 39.5% of convenience stores sold bananas, 35.7% sold apples, 29.6% sold tomatoes, 25.9% sold oranges, 25.9% sold watermelon, and 14.8% sold cucumbers. Less than 10% of convenience stores sold carrots, broccoli, or corn. Approximately 60% of convenience stores sold whole grain bread, whereas 95.8% sold white bread. While 39% of convenience stores sold baked potato chips, 96.4% sold regular potato chips. When comparing healthier food availability by ZIP Code, a significantly larger proportion of convenience stores in ZIP B sold bananas (*χ* ^2^ = 4.17, *P* = 0.041), watermelon (*χ* ^2^ = 7.92, *P* = 0.005), whole grain bread (*χ* ^2^ = 8.33, *P* = 0.004), and baked potato chips (*χ* ^2^ = 13.68, *P* < 0.001).

Table 3 compares the cost of items sold at convenience stores by ZIP Code. On average, convenience stores in ZIP B sold healthier foods like diet cola (*t* = −2.12, *P* = 0.044), bananas (*t* = −3.10, *P* = 0.015), oranges (*t* = −3.49, *P* = 0.018), tomatoes (*t* = −2.89, *P* = 0.010), broccoli (*t* = −9.62, *P* < 0.001), and cucumbers (*t* = −8.21, *P* < 0.001) at significantly higher prices compared to the same items sold at convenience stores in ZIP A. On average, convenience stores in ZIP B also sold white bread at significantly higher prices than convenience stores in ZIP A (*t* = −3.70, *P* = 0.001).

## 4. Discussion

While disparities in food culture have been well examined as it pertains to food access, quality, and price in grocery stores, less research has investigated differences in the food culture of convenience stores by residential affluence and ethnic composition. The American Diabetes Association (ADA) often gives recommendations for purchasing healthier foods at grocery stores and places little emphasis on those found in convenience stores, despite their role in shaping the food culture in rural and less affluent areas. Investigating the availability and affordability of healthy foods in convenience stores is especially relevant in today's high-paced “grab-and-go” society, where the snacks consumed between meals have a great potential to influence eating patterns conducive to or protective against chronic conditions, including heart disease and diabetes.

We conducted a nutritional food environment assessment to measure food culture in convenience stores based on nutritional standards, food availability, and food costs in two economically diverse ZIP Codes located in Bexar County, Texas. When comparing food availability by ZIP Code, a significantly larger proportion of convenience stores located within the more affluent neighborhoods (ZIP B) tended to have healthier items that were readily available, including certain fruits, whole grain bread, and baked potato chips when compared to convenience stores located within the less affluent comparison neighborhoods (ZIP A). In terms of price, food costs on selected items were generally higher in ZIP B than in ZIP A.

In an urban food store study conducted by Farley and colleagues [33], findings indicate that all grocery stores sold fresh fruits and vegetables compared to only about 10% of convenience stores. This study also reported that, while convenience and other small food stores offered the least healthy mix of items, all types of food stores allotted more shelf space to unhealthy food choices than to healthy items. In a similar study, Bustillos and colleagues [34] documented the availability of staple foods in two rural Texas counties. Findings from this study support those conducted in urban areas where traditional food stores, like grocery stores, offered a better variety of healthy foods like meats, reduced-fat and skim milk, and whole grains.

Along with the availability of fresh and healthful foods, high prices of convenience store items present an additional barrier. Studies that assessed the shelf space within grocery stores and other types of food stores reported higher prices at convenience stores than grocery stores for similar food items [12, 35, 36]. In a study conducted by the United States Department of Agriculture (USDA), researchers examined the prices of three staple healthy food items at convenience and grocery stores using Nielsen Homescan panel data [12]. The most popular items bought by panelists were fluid milk, ready-to-eat cereal, and bread. Results from this study indicate that the price per ounce for all three items is significantly higher in convenience stores when compared to grocery stores. Similar studies support findings that the price of goods is often higher at convenience stores [37–39].

As in Bexar County, Texas, and other regions of the United States, marketing strategies often drive studies about the purchasing of nutritional foods. Consumers consider three aspects before purchasing a food item: perceived value, perceived nutrition, and taste [40]. However, a multitude of other factors, including income, also influence the consumer's primary motive for purchase. Marketing analysts utilize price reduction strategies, which aim to promote the selection of target foods by lowering their cost comparatively to alternative food items [40]. According to the USDA, food-at-home expenditure had its lowest annual increase since 1967 of 0.3 percent, while food-away-from-home expenditures rose 1.3 percent in 2010 [41]. With readily available food options at fast food restaurants and convenience stores, residents are more likely to purchase food items at these locations, although they lack healthier options when compared to grocery stores. In a price reduction intervention study of 12 worksites and 12 secondary schools in Minnesota, French and colleagues [42] found that the price reduction significantly increased sales of low-fat snack items. Although it is suggested that these pricing strategies can promote healthy eating if healthier options are targeted [40, 42], it is ultimately the consumer who has the choice to decide the final purchased product; however, consumers lack the opportunity to purchase these items without the access to healthy foods.

### 4.1. Limitations

This study has limitations, which should be acknowledged. First, not all convenience stores were audited in the two ZIP Codes included in this study, thus these stores surveyed may not be fully representative of all convenience stores in these areas. Second, despite the use of a validated audit instrument used to assess food inventory in the convenience stores, the individuals who performed the audits differed; therefore, the consistency or interrater reliability of the inventory documentation between study investigators was unknown. Third, while this study investigated the presence and price of food items in convenience stores, we did not assess the actual food being purchased by the residents in these areas (either healthy or unhealthy). Consequently, the purchasing decisions of the community were unknown. Further, neither the nutritional content nor compositions of the foods inventoried were assessed, which limited our ability to confirm the actual healthiness of the foods offered in the convenience stores. Fourth, although audits included quality ratings of available foods in the convenience stores, important details about food quality such as product expiration dates or damaged packaging were not specifically collected. Fifth, this study did not capture the health status, diagnoses, risk factors, or financial stability of the convenience store patrons which made it difficult to determine if these patrons required healthier food choices. Not knowing the characteristics of the convenience store patrons hindered our ability to know if they could visit other stores to get healthier food items or afford the items they wanted to purchase. Lastly, it was unknown as to what influenced patrons' purchases (e.g., health, cost, proximity to residence, or other factors).

## 5. Conclusion

This study is considered an important step in increasing our understanding of the role convenience stores can play in positively shaping a community's food environment. Our study demonstrates that while food categories can be nutritionally delineated between “healthy” and “unhealthy” based on national ADA guidelines, such decisions are often tethered to food outlet's operational and financial circumstances and decision making. This results in food choices and availability that may actually be a reflection of a community's socioeconomic position. Amid our study's limitations, the etiological understanding of a community's health status can be better understood by how food outlets, such as convenience stores, can potentially shape and determine the nutritional habits of neighborhood residents. In this specific case, future studies are needed to investigate the relationships between the ethnic composition of communities, residential affluence, and food culture indicators. Such investigations may become critical to improve the food culture of convenience stores and track purchasing and diabetes trends associated with stocking healthier foods at affordable prices.

## Figures and Tables

**Table 1:** Bexar County characteristics by ZIP Code.

	ZIP Code		Bexar County
	78207 (ZIP A)	78240 (ZIP B)	
Total population	55,514	51,111	1,392,931
Hispanic population (percent of total population)	50,435 (90.9%)	24,358 (47.7%)	757,033 (54.4%)
Median household income ($1 K increments)	20.12	39.63	38.33
Median age (years)	30.10	31.50	32.10
Less than a high school education (percent of residents aged 25+)	15,293 (47.3%)	2,535 (7.6%)	17,828 (27.1%)
Total deaths (in 2009)	480	352	10,506
Diabetes-related deaths (percent of total deaths)	23 (4.8%)	11 (3.1%)	337 (3.2%)
Heart disease-related deaths (percent of total deaths)	114 (23.8%)	80 (22.7%)	2,285 (21.8%)
Number of convenience stores	23	14	685
Number of grocery stores (excluding convenience stores)	14	4	160
Number of restaurants	50	55	2,339

**Table 2:** Availability of beverages, produce, and grains in convenience stores.

	Zip Code		Total	*X* ^2^	*P*
	78207 (ZIP A)	78240 (ZIP B)			
	(*n* = 19)	(*n* = 9)			
Beverages					
Milk: low fat, skim, or 1% (half gallon)	2 (10.5%)	1 (11.1%)	3 (10.7%)	0.002	0.963
Milk: 2% (half gallon)	5 (35.7%)	8 (88.9%)	13 (59.1%)	8.703	0.003
Diet cola (20 oz)	19 (100.0%)	9 (100.0%)	28 (100.0%)	—	—
Regular cola (20 oz)	19 (100.0%)	9 (100.0%)	28 (100.0%)	—	—
100% juice (no sugar added: 15.2 oz)	13 (68.4%)	7 (77.8%)	20 (71.4%)	0.262	0.609
Juice drink (sugar added: 15.2 oz)	6 (31.6%)	5 (55.6%)	11 (39.3%)	1.472	0.225
Fruits and vegetables					
Bananas	5 (26.3%)	6 (66.7%)	11 (39.3%)	4.169	0.041
Apples	5 (26.3%)	5 (55.6%)	10 (35.7%)	2.274	0.132
Oranges	3 (15.8%)	4 (50.0%)	7 (25.9%)	3.431	0.064
Watermelon	2 (10.5%)	5 (62.5%)	7 (25.9%)	7.919	0.005
Carrots	2 (10.5%)	0 (0.0% )	2 (7.4%)	0.909	0.340
Tomatoes	7 (36.8%)	1 (12.5%)	8 (29.6%)	1.600	0.206
Broccoli	1 (5.3%)	1 (12.5%)	2 (7.4%)	0.430	0.512
Corn	2 (10.5%)	0 (0.0%)	2 (7.4%)	0.909	0.340
Cucumbers	3 (15.8%)	1 (12.5%)	4 (14.8%)	0.048	0.826
Grains					
Whole grain bread	3 (30.0%)	7 (77.8%)	10 (58.8%)	8.330	0.004
White bread	15 (93.8%)	8 (88.9%)	23 (95.8%)	0.522	0.470
Liquid oils					
Baked potato chips	3 (15.8%)	8 (88.9%)	11 (39.3%)	13.682	<0.001
Regular potato chips	18 (94.7%)	9 (100.0%)	27 (96.4%)	0.491	0.483

Some percentages slightly vary due to missing data for certain products.

**Table 3:** Affordability of beverages, produce, and grains in convenience stores.

	Zip Code		*t*	*P*
	78207 (ZIP A)	78240 (ZIP B)		
	(*n* = 19)	(*n* = 9)		
Beverages				
Milk: low fat, skim, or 1% (half gallon)	3.19 (±0.28)	2.29 (±0.42)	2.496	0.130
Milk: 2% (half gallon)	3.29 (±0.27)	3.14 (±1.30)	0.197	0.852
Diet cola (20 oz)	1.35 (±0.13)	1.45 (±0.05)	−2.117	0.044
Regular cola (20 oz)	1.37 (±0.11)	1.45 (±0.05)	−1.971	0.060
100% juice (no sugar added; 15.2 oz)	1.66 (±0.19)	2.00 (±0.58)	−1.923	0.071
Juice drink (sugar added: 15.2 oz)	1.55 (±0.15)	1.63 (±0.23)	−0.725	0.487
Fruits and vegetables				
Bananas	0.43 (±0.15)	1.13 (±0.43)	−3.104	0.015
Apples	1.12 (±0.94)	1.24 (±0.53)	−0.239	0.818
Oranges	0.45 (±0.09)	0.90 (±0.21)	−3.488	0.018
Watermelon	1.99 (±0.00)	2.19 (±1.09)	−0.171	0.873
Carrots	0.09 (±0.27)	—	—	—
Tomatoes	0.18 (±0.27)	0.99 (±0.00)	−2.885	0.010
Broccoli	0.07 (±0.30)	2.99 (±0.00)	−9.624	<0.001
Corn	0.10 (±0.32)	—	—	—
Cucumbers	0.14 (±0.34)	2.99 (±0.00)	−8.208	<0.001
Grains				
Whole grain bread	2.01 (±0.77)	2.83 (±0.17)	−1.831	0.203
White bread	2.06 (±0.39)	2.65 (±0.32)	−3.695	0.001
Liquid oils				
Baked potato chips	0.99 (±0.00)	0.99 (±0.00)	—	—
Regular potato chips	2.22 (±1.43)	2.32 (±1.58)	−0.169	0.868

*Means and standard deviations presented in U.S. dollars.
